# Combination Therapy of Radiofrequency Ablation and Transarterial Chemoembolization for Unresectable Hepatocellular Carcinoma: A Retrospective Study

**DOI:** 10.1097/MD.0000000000003754

**Published:** 2016-05-20

**Authors:** Chengwu Tang, Jian Shen, Wenming Feng, Ying Bao, Xiaogang Dong, Yi Dai, Yinyuan Zheng, Jianping Zhang

**Affiliations:** From the Department of General Surgery, The Second Affiliated Hospital, Nanjing Medical University, Nanjing (CT, JS, XD, YD, JZ) and Departments of General Surgery (CT, WF, YB) and Radiology (YZ), First People's Hospital Affiliated to Huzhou University Medical College, Huzhou, China.

## Abstract

The treatment efficacy of unresectable hepatocellular carcinoma (HCC) is still not promising. This study aimed to compare the efficacy and safety of radiofrequency ablation (RFA) combined with transarterial chemoembolization (TACE) for unresectable HCC with a single treatment.

Between June 2009 and June 2012, 132 patients who were diagnosed with unresectable HCC and accepted nonsurgical treatments in our center were enrolled in this retrospective study. On the basis of treatment modality, they were allocated to 3 groups: 49 patients accepted RFA (RFA group); 43 patients accepted TACE (TACE group); and 40 patients accepted RFA following TACE (combination group). Clinical data including complications, treatment success rate, hospitalization costs, intrahepatic recurrence-free survival, overall survival, and factors influencing survival were retrospectively analyzed.

Patient characteristics between these groups showed no significant difference. Treatment success was achieved in all patients of 3 groups. The combination group had a significantly higher total hospitalization cost to treatment than the TACE group (63,708.14 ± 9193.81 Chinese yuan vs 37,534.88 ± 6802.84 Chinese yuan; *P* = 0.0000). All complications were controllable and no permanent adverse sequelae or procedure-related deaths were observed. The 3-year intrahepatic recurrence-free survival probability was significantly better in the combination group than in the TACE group (42.50% vs 20.93%; hazard ratio [HR], 0.5105; 95% confidence interval [CI], 0.3022–0.8625; *P* = 0.0094) or the RFA group (42.50% vs 22.45%; HR, 0.5233; 95% CI, 0.3149–0.8697; *P* = 0.0111).The 3-year overall survival probability was significantly better in the combination group than in the TACE group (45.00% vs 26.53%; HR, 0.5069; 95% CI, 0.2936–0.8752; *P* = 0.0100) or the RFA group (45.00% vs 27.91%; HR, 0.4913; 95% CI, 0.2928–0.8246; *P* = 0.0054). Main tumor size, number of tumors, and treatment modality were demonstrated to be important factors associated with 3-year intrahepatic recurrence-free survival probability and overall survival probability (*P* < 0.05) by univariate and multivariate analyses.

Combination therapy of RFA and TACE was superior to TACE alone or RFA alone in improving survival for patients with unresectable HCC.

## INTRODUCTION

Hepatocellular carcinoma (HCC) has become 1 of the world's most common primary malignancies and leads to over 500,000 deaths each year globally.^1^ Most HCC are diagnosed at intermediate-advanced stage,^2^ and only approximately 30% of patients are suitable for radical therapies.^3^ Therefore, the long-term survival of patients with intermediate-advanced stage HCC remains unfavorable.

Transarterial chemoembolization (TACE) has been established as the standard therapy for patients with unresectable HCC. TACE slows tumor progression and improves survival by combining the effect of targeted chemotherapy with that of ischemic necrosis induced by arterial embolization.^4^ The survival benefit of TACE treatment has been proved by several randomized clinical trial.^5,6^ However, it is difficult to achieve complete necrosis of liver tumor with TACE alone therefore tumor relapse after TACE is universal.^7^ Recently, radiofrequency ablation (RFA) has been shown safe and successful for local tumor control in patients with HCC and is increasingly used for patients with HCC.^8^ Previous studies have shown that RFA can achieve complete necrosis in more than 90% of small HCCs.^9,10^ Nevertheless, the complete necrosis rate of RFA treatment in patients with advanced or large HCC is unsatisfactory, even if the procedure is repeated.^11^

The limitations of TACE and RFA will lead to inadequate control of HCC tumors.^12^ Consequently, multimodal treatment is an appealing alternative, especially for patients with large and unresectable HCC. The aim of this study was to assess the efficacy and safety of combination therapy of RFA and TACE for unresectable HCC.

## METHODS

### Patients

Between June 2009 and June 2012, 132 patients with unresectable HCC who underwent nonsurgical treatments in First People's Hospital affiliated to Huzhou University Medical College were enrolled in this retrospective study. The diagnosis of HCC was established by either histopathology or typical appearance of HCC on 2 sets of imaging studies (ultrasonography, computed tomography [CT], angiography, and magnetic resonance imaging) and based on high plasma levels of a serum alpha-fetoprotein (AFP) value exceeding 400 ng/mL.^13^ Eligibility criteria were as follows: age ≤ 75 years, Karnofsky performance score (KPS) ≥ 70; no indication for resection; 3 or fewer lesions, each larger than 3 cm but ≤10.0 cm in greatest diameter; lesions located at least 0.5 cm away from the hepatic hilum or gallbladder and the common bile duct and 1 cm from the bowel; no previous HCC treatment; liver function of Child-Pugh A–B; no portal vein thrombosis or extrahepatic metastasis; no diffuse or infiltrative tumors; no refractory ascites or renal failure and complete clinicopathologic and follow-up data.

On the basis of treatment modality, these patients were allocated to 3 groups. In the RFA group, 49 patients accepted ultrasound-guided percutaneous RFA as the sole first-line anticancer therapy. In the TACE group, 43 patients accepted TACE as the sole first-line anticancer therapy. And in the combination group, RFA was administrated within 1 to 2 weeks following 2 sessions of TACE in 40 patients.

This study was approved by the Institutional Review Board of First People's Hospital affiliated to Huzhou University Medical College. Written informed consent was obtained from all patients. All procedures performed in studies involving human participants were in accordance with the ethical standards of the institutional and/or national research committee and with the 1964 Helsinki declaration and its later amendments or comparable ethical standards.

### TACE Procedure

TACE was administrated in the combination group and TACE group using the Seldinger technique.^14^ Tumor feeding artery was identified and catheterized after hepatic arteriography; 5-fluoruracil (1000 mg/m^2^) and cisplatin (80 mg/m^2^) were slowly injected into the selected feeding artery through the catheter. Then, the artery was embolized with the mixture of 5 to 30 mL lipiodol and mitomycin-C (6 mg/m^2^) emulsion. Afterwards, additional embolization with gelatin sponge was performed for lesions larger than 5 cm or with abundant blood supply. After TACE procedure, patients were supervised carefully, and analgesia (pentazocine or meperidine) was administered when necessary. TACE was usually conducted about every 4 weeks and each session of TACE required hospitalization for 7 to 10 days. When serious bone marrow depression, liver function impairment or other major complications were observed after TACE procedure, discharge was delayed, and chemoembolization regimen was adjusted accordingly.

### RFA Procedure

RFA was administrated in the combination group and RFA group about every 2 weeks. Patients from the combination group received RFA within 1 to 2 weeks following 2 sessions of TACE. Enhanced CT was utilized to determine the efficacy of TACE procedure and then the detected lesions were treated by RFA. A commercially available RF ablation system (S-1500 Radiofrequency Ablation System; Medsphere International, Shanghai, China) was used to generate up to150 W of energy to cause adequate coagulation necrosis of the target tissue and a 0.5 to 1.0 cm margin. Expandable electrodes (Medsphere International, Shanghai, China) with an outer insulated needle and a core needle with multiple small umbrella-shaped electrodes were advanced under ultrasound guidance. The core needles were expanded and retracted by a movable handle and the diameter at expansion ranged from 3.0 to 5.0 cm. Selecting an electrode diameter size depends on the size of the target tumor. Treatment began at a 50 W level, with wattage increasing 10 W every 2 min until tissue impedance increased and the prevention of further current flow or for 10 min. During the procedure, we utilized real-time ultrasound to monitor echo changes in the treatment area. For some larger lesions, additional overlapping RF ablation treatments were performed when needed to attain adequate ablation margins by changing in radial position of umbrella-shaped electrodes. Track ablation was conducted during withdrawing the electrode in all patients. The RFA procedure was performed after intravenous administration of 2.5 to 5.0 mg of midazolam and 0.05 to 0.10 mg of fentanyl as well as local anesthesia (5–15 mL of 1% lidocaine).

During RFA procedure, patients’ vital signs were continuously supervised. All patients accepted ultrasound scan to detect active bleeding before being transferred to an inpatient ward within 2 to 4 h after RFA procedure. Conventionally, patients stayed in hospital for 1 to 3 days after RFA procedure and hospital stay was prolonged in patients with large tumors, organ failures, or major complications. No prophylactic antibiotic was administrated before or after RFA procedure.

### Assessment and Follow-Up

The treatment success was assessed by enhanced CT within 2 weeks after TACE and within 1 week after RFA, respectively. The termination of treatment was based on 2 criteria: insufficient liver function after treatment or no residue tissue in the liver detected by follow-up imaging. After last treatment session, patients were assessed every 3 months for 2 years and every 6 months thereafter by enhanced CT, ultrasonography, serum biochemistry, and clinical examination. Total hospitalization cost to treatment was comprised of hospitalization cost of each treatment session. Patients were followed up until last follow-up or death. The primary end point was overall survival and the secondary end point was intrahepatic recurrence-free survival. The complications were also assessed. A major complication was defined as an event that led to substantial morbidity and disability, increased the level of care required, resulted in hospital admission or substantial lengthening of hospitalization. All other complications were considered minor.^15^ Overall survival was measured from the date of treatment initiation to death or the date of last follow-up. Intrahepatic recurrence-free survival was measured from the date of treatment initiation to intrahepatic recurrence, death or last follow-up, whichever came first. Intrahepatic recurrence was defined as new lesions distant from the initial tumors or local tumor recurrence and was diagnosed on the basis of imaging and, if necessary, cytologic analysis or biopsy. According to our ethical committee, all the patients with relapsing or progressive tumors were treated with the best possible options (such as repeated TACE or RFA, supportive care, etc.).

### Statistical Analysis

All analyses were by intention to treat. Pearson χ^2^ tests with Fisher exact probability were performed to compare the frequency distributions of categorical variables between groups. One-way analysis of variance was used to test the differences in means between groups for continuous variables. Overall survival and intrahepatic recurrence-free survival were analyzed by the Kaplan–Meier method and survival curves were compared by the log-rank test. A Cox proportional hazards model was used to identify independent clinical factors or groups that influenced survival. All statistical analyses were carried out with statistical software package SPSS for Windows (ver. 17.0, SPSS, Chicago, IL). All reported *P* values are 2-sided, with *P* < 0.05 considered statistically significant.

## RESULTS

### Patient Characteristics

The baseline characteristics and indications of the patients were summarized in Table 1. No significant difference was found in age, gender, KPS and AFP level, Child-Pugh class, number of tumors, main tumor size, and background liver disease among the 3 groups.

**TABLE 1:**
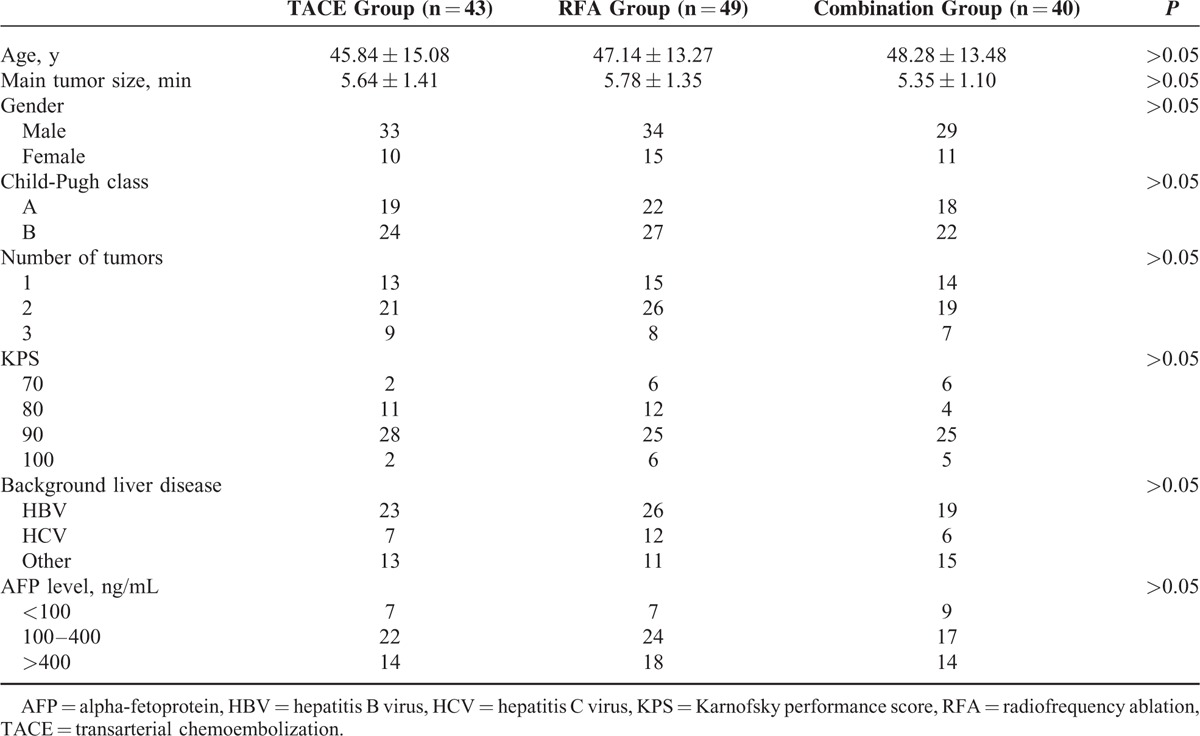
Patient Characteristics

### Treatment Success and Hospitalization Cost

Tumor enhancement disappeared in all 132 patients (treatment success rate, 100%). Treatment success was achieved after 3 TACE sessions in 24 patients and 4 TACE sessions in 19 patients in the TACE group with a total hospitalization cost of 37,534.88 ± 6802.84 Chinese yuan. In the RFA group, treatment success was achieved after 3 RAF sessions in 25 patients and 4 RAF sessions in 24 patients with a total hospitalization cost of 62,816.33 ± 9091.37 Chinese yuan. In the combination group, treatment success was achieved after 2 RAF sessions in 20 patients, 3 RAF sessions in 14 patients and 4 RAF sessions in 6 patients following 2 TACE sessions with a total hospitalization cost of 63,708.14 ± 9193.81 Chinese yuan. Therefore, the combination group had a significantly higher total hospitalization cost than the TACE group (*P* = 0.0000). There was no significant difference in total hospitalization cost between the combination group and the RFA group (*P* = 0.6481).

### Complications

There was no treatment-related death observed. Moderate intraperitoneal hemorrhage occurred in 3 patients after RFA (2 cases from the RFA group and 1 case from the combination group) and was controlled by nonsurgical treatment. A total of 49 patients experienced a mild–moderate degree of bone marrow depression (26 cases from the TACE group and 23 cases from the combination group). One patient from the combination group developed hemothorax and percutaneous drainage was performed. Most patients developed minor complications including nausea, fever, post-treatment abdominal pain, and transient liver function injury but none required medical intervention.

### Intrahepatic Recurrence-Free Survival

**FIGURE 1:**
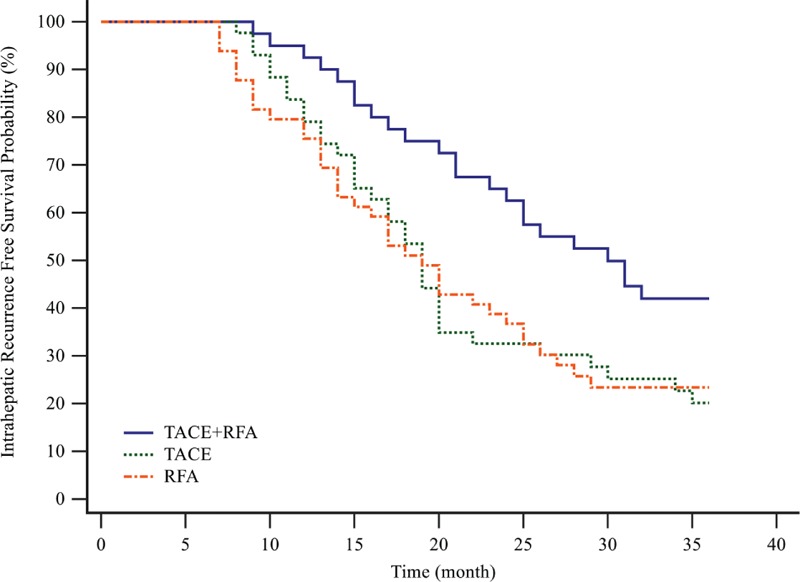
Kaplan–Meier curves of 3-y intrahepatic recurrence-free survival stratified by treatment modality. During the 1st 3 y after treatment initiation, 34 of 43 patients in the TACE group, 38 of 49 patients in the RFA group, and 23 of 40 patients in the combination group had intrahepatic recurrence. The 3-y intrahepatic recurrence-free survival probability was significantly better in the combination group than in the TACE group (HR, 0.5105; 95% CI, 0.3022–0.8625; *P* = 0.0094 by log-rank test) or the RFA group (HR, 0.5233; 95% CI, 0.3149–0.8697; *P* = 0.0111 by log-rank test). CI = confidence interval, HR = hazard ratio, RFA = radiofrequency ablation, TACE = transarterial chemoembolization.

During the 1st 3 years after treatment initiation, 34 of 43 patients in the TACE group, 38 of 49 patients in the RFA group, and 23 of 40 patients in the combination group had intrahepatic recurrence. The probabilities of intrahepatic recurrence-free survival at 3 years were 20.93% in the TACE group, 22.45% in the RFA group, and 42.50% in the combination group. The 3-year intrahepatic recurrence-free survival probability was significantly better in the combination group than in the TACE group (hazard ratio [HR], 0.5105; 95% confidence interval [CI], 0.3022–0.8625; *P* = 0.0094 by log-rank test) or the RFA group (HR, 0.5233; 95% CI, 0.3149–0.8697; *P* = 0.0111 by log-rank test; Figure 1).

The results of univariate and multivariate analyses of factors that influenced 3-year intrahepatic recurrence are summarized in Table 2. In the univariate analysis, main tumor size (*P* = 0.0234), number of tumors (*P* = 0.0004), and treatment modality (*P* = 0.0243) significantly correlated with 3-year intrahepatic recurrence probability. In the multivariate analysis, these 3 factors were demonstrated to be significantly independent factors for 3-year intrahepatic recurrence (*P* < 0.05).

**TABLE 2:**
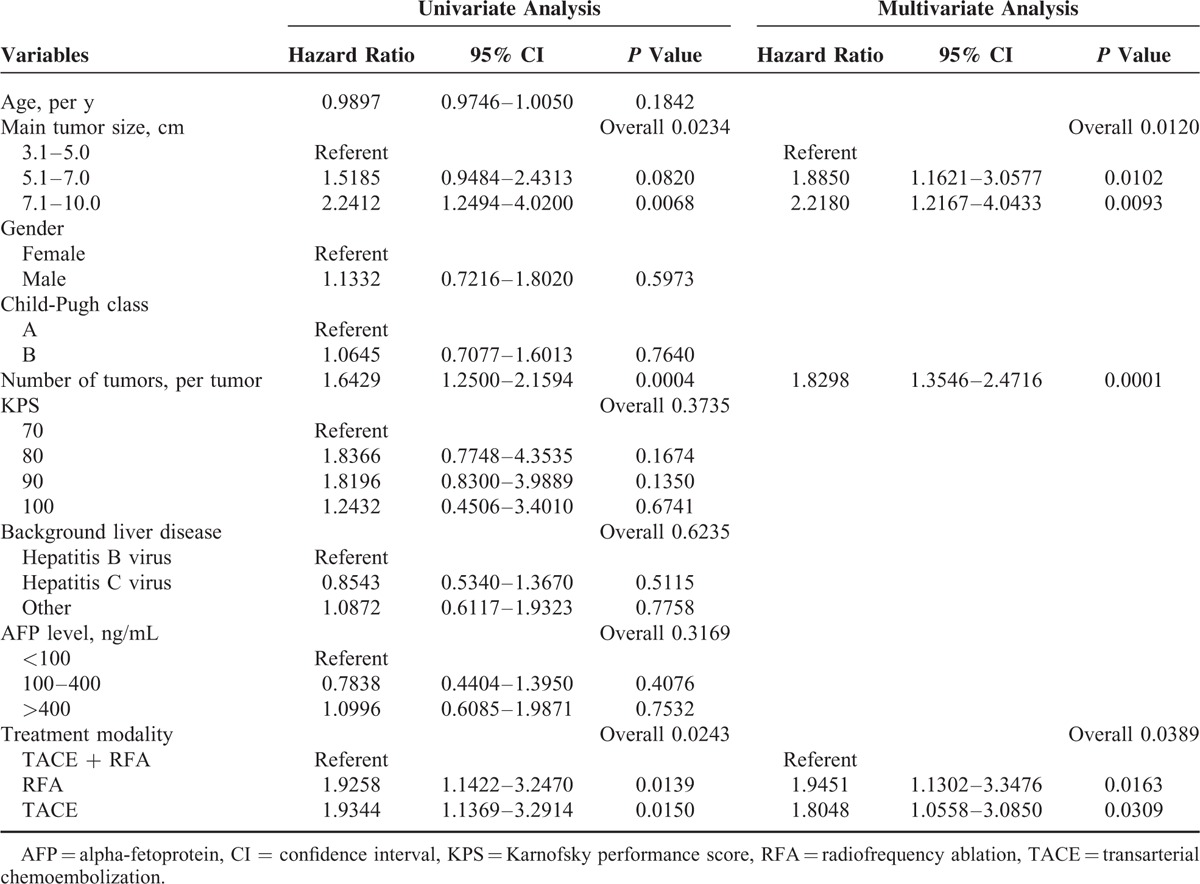
Univariate and Multivariate Analyses of Factors That Influenced Intrahepatic Recurrence-Free Survival

### Overall Survival

**FIGURE 2:**
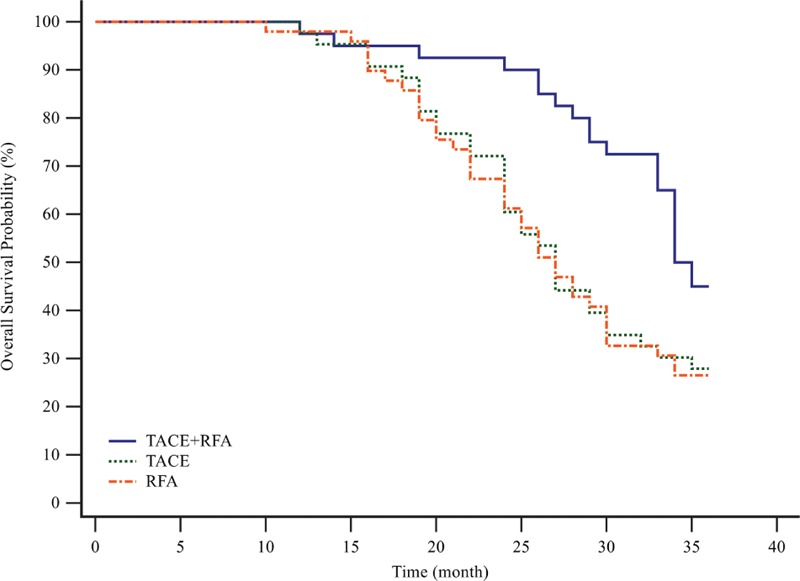
Kaplan–Meier curves of 3-y overall survival stratified by treatment modality. During the 1st 3 y after treatment initiation, 31 of 43 patients in the TACE group, 36 of 49 patients in RFA group, and 22 of 40 patients in the combination group were dead. The 3-y overall survival probability was significantly better in the combination group than in the TACE group (HR, 0.5069; 95% CI, 0.2936–0.8752; *P* = 0.0100 by log-rank test) or the RFA group (HR, 0.4913; 95% CI, 0.2928–0.8246; *P* = 0.0054 by log-rank test). CI = confidence interval, HR = hazard ratio, RFA = radiofrequency ablation, TACE = transarterial chemoembolization.

During the 1st 3 years after treatment initiation, 31 of 43 patients in the TACE group, 36 of 49 patients in the RFA group, and 22 of 40 patients in the combination group were dead. Respectively, 19 patients in the TACE group, 16 patients in the RFA group, and 11 patients in the combination group died of progression of HCC. Other deaths were caused by hepatic failure. The probabilities of overall survival at 3 years were 27.91% in the TACE group, 26.53% in the RFA group, and 45.00% in the combination group. The 3-year overall survival probability was significantly better in the combination group than in the TACE group (HR, 0.5069; 95% CI, 0.2936–0.8752; *P* = 0.0100 by log-rank test) or the RFA group (HR, 0.4913; 95% CI, 0.2928–0.8246; *P* = 0.0054 by log-rank test; Figure 2).

The results of univariate and multivariate analyses of factors that influenced 3-year overall survival are summarized in Table 3. In the univariate analysis, main tumor size (*P* = 0.0321), number of tumors (*P* = 0.0008), and treatment modality (*P* = 0.0207) significantly correlated with 3-year overall survival probability. In the multivariate analysis, these 3 factors were demonstrated to be significantly independent factors for 3-year overall survival (*P* < 0.05).

**TABLE 3:**
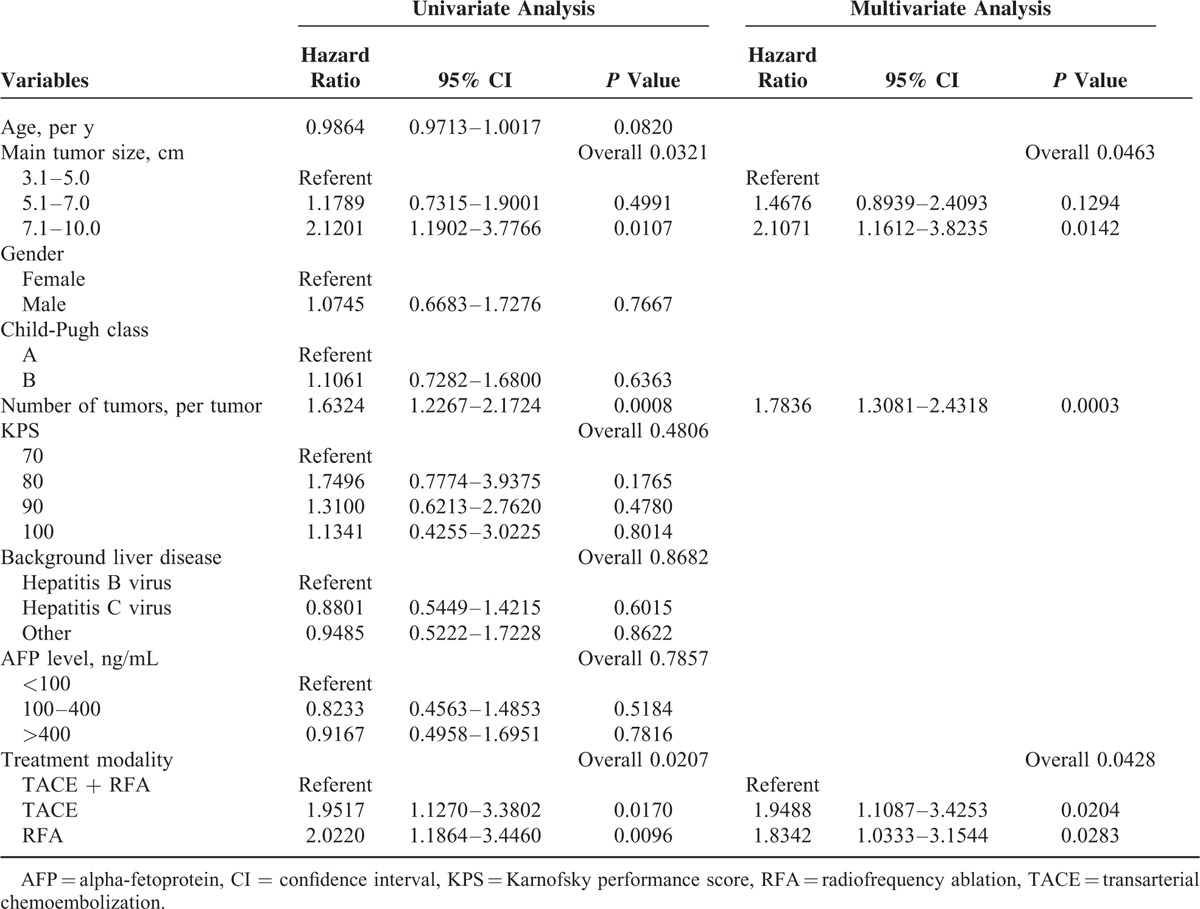
Univariate and Multivariate Analyses of Factors That Influenced Overall Survival

## DISSCUSSION

HCC has become of the most common primary malignancies in China, with the incidence rate up to 80 per million of the populations. It is known that only 30% of patients with early stage HCC may benefit from radical therapies and the 5-year recurrence rate after resection reaches above 70%.^16^ To achieve better long-term survival of patients with advanced HCC, the most important issues are the selection of appropriate modalities against unresectable HCC and prevention of intrahepatic recurrence after initial therapies. RFA once considered as an alternative treatment to partial hepatectomy for HCC with impaired liver function has been widely utilized as a first-line therapy for small HCC.^17^ TACE is also well accepted as a palliative therapy for unresectable HCC for its minimal invasiveness and efficiency in tumor eradication.^18^ However, both TACE and RFA have some inherent shortages which lead to inadequate necrosis of HCC tumors larger than 3 cm.^4^ Therefore, combination therapy is a promising alternative, especially for patients with large or unresectable HCC. Because blood flow to the tumor leads to heat loss which may bring down the curative effectiveness of RFA, a possible way to increase the ablation size of RFA thermal lesions would be to reduce or eliminate the heat loss that is mediated by tissue perfusion.^19^ Theoretically, embolization of hepatic arterial flow and peripheral portal vein around the tumor by TACE may effectively increase RFA effectiveness.^20^ Moreover, as a regional therapy, TACE can detect satellite tumors surrounding the zone of RFA-induced necrosis missed by imaging examination. Therefore, combination therapy of TACE and RFA is increasingly utilized to treat patients with advanced HCC.

Our study suggested that combination therapy of TACE and RFA obtains better long-term survival than either single treatment in unresectable HCC. The 3-year intrahepatic recurrence-free survival probability was significantly higher in the combination group than in the TACE group (HR, 0.5105; 95% CI, 0.3022–0.8625; *P* = 0.009) or the RFA group (HR, 0.5233; 95% CI, 0.3149–0.8697; *P* = 0.0111). Moreover, the 3-year overall survival probability was also significantly higher in the combination group than in the TACE group (HR, 0.5069; 95% CI, 0.2936–0.8752; *P* = 0.0100) or the RFA group (HR, 0.4913; 95% CI, 0.2928–0.8246; *P* = 0.0054). These results could be ascribed to blockaded tissue blood flow to the tumor, decreased tissue impedance, and disrupted intratumoral septa caused by TACE procedure.^21–23^ Therefore, more effective ablation was achieved safely during the RFA procedure following TACE procedure.^19^

Univariate and multivariate analyses showed that tumor number was associated with poor 3-year intrahepatic recurrence-free survival probability and overall survival probability as described in previous reports.^22^ Results of our study also show that, in addition to tumor number, main tumor size and treatment modality were significant factors that affect 3-year intrahepatic recurrence-free survival and overall survival.

We found that treatment success was achieved with low complication rates in all patients of 3 groups. The severest complication encountered was moderate intraperitoneal hemorrhage after RFA in the combination group and RFA group, and was controlled with conservative treatment. All complications were controllable and no permanent adverse sequelae or procedure-related deaths were observed. Thus combination therapy of TACE followed by RFA appears to be relatively safe. We also found that due to the costly single-use disposable electrodes utilized during RFA procedure, the combination group had a significantly higher total hospitalization cost to treatment than the TACE group. However, the increase in cost was acceptable as a result of significantly improved survival rates.

In conclusion, this retrospective study found that combination therapy of RFA and TACE was superior to TACE alone or RFA alone in improving 3-year intrahepatic recurrence-free survival and 3-year overall survival for patients with unresectable HCC. However, because of the retrospective nature and the small sample size, further prospective study with large sample size is needed to confirm the results of our study.
